# Chemical Exchange Saturation Transfer Magnetic Resonance Imaging (CEST MRI) in Lung Cancer: A Narrative Review of Translational Challenges and Clinical Potential

**DOI:** 10.7759/cureus.102263

**Published:** 2026-01-25

**Authors:** Saurabh Dubey

**Affiliations:** 1 Internal Medicine, State University of New York Downstate Health Sciences University, Brooklyn, USA

**Keywords:** cest, cest-mri, chemical exchange, lung tumor, mri

## Abstract

Chemical exchange saturation transfer magnetic resonance imaging (CEST MRI) is a sophisticated molecular imaging technique that facilitates the indirect detection of low-concentration endogenous metabolites through their exchange with bulk water. By applying a selective radiofrequency (RF) saturation pulse to labile protons - such as those found in amide, amine, and hydroxyl groups - CEST MRI generates a measurable reduction in the water signal. This sensitivity allows for the non-invasive assessment of critical physiological variables, including pH, protein concentration, and metabolite levels. In the context of oncology, specific applications such as amide proton transfer (APT) and acidoCEST have emerged as powerful tools for probing the hallmarks of malignancy, such as tissue acidosis and altered protein expression, which are often invisible to conventional anatomical imaging.

Despite the historical challenges of thoracic MRI, including respiratory motion and susceptibility artifacts at air-tissue interfaces, advancements in hardware and pulse sequences have paved the way for CEST applications in lung imaging. Lung cancer remains a leading cause of global cancer mortality, necessitating more nuanced diagnostic tools to differentiate between malignant and benign lesions and to monitor therapeutic response at a biochemical level. This narrative review explores the fundamental principles and current research regarding CEST MRI in lung malignancies. By providing a molecular-level characterization of the tumor microenvironment, CEST MRI holds the potential to serve as a functional biomarker, enhancing the precision of early diagnosis, tumor subtyping, and longitudinal management of lung cancer patients.

## Introduction and background

Introduction

Chemical exchange saturation transfer magnetic resonance imaging (CEST MRI) is a novel molecular imaging technique that provides functional and metabolic information beyond conventional anatomical imaging. It achieves this by exploiting the exchange of protons between water and endogenous or exogenous metabolites containing labile protons, such as amide (-NH), amine (-NH₂), and hydroxyl (-OH) groups. These exchangeable protons resonate at specific frequencies distinct from water, allowing selective saturation via a radiofrequency (RF) pulse. When these saturated protons exchange with water protons, a reduction in the water signal occurs, which can be detected and quantified, generating contrast sensitive to specific biochemical environments [1-4].

CEST MRI is uniquely positioned among MRI modalities because it enables the indirect detection of low-concentration biomolecules without the need for traditional contrast agents in some applications. CEST MRI is a broad term that encompasses various techniques. Among the different types of CEST imaging, amide proton transfer (APT) and acidoCEST MRI have gained prominence for their abilities to provide insight into tissue acidity and protein content, respectively. These characteristics are particularly relevant to oncology, where changes in the tumor microenvironment - such as acidosis and altered protein expression - are hallmarks of malignancy [4,5].

Despite its growing application in neurological and abdominal imaging, the use of CEST MRI in thoracic imaging, particularly for lung malignancies, remains an emerging area of research. Lung cancer is the leading cause of cancer-related mortality worldwide, and early, accurate diagnosis remains critical for improving patient outcomes. Conventional imaging modalities, such as computed tomography (CT) and positron emission tomography (PET), are routinely used for detection, staging, and monitoring [6]. Accurate imaging is essential in the staging, initial diagnosis, and management of lung malignancies. However, these modalities primarily provide anatomical or metabolic information and are often limited in their ability to differentiate between malignant and benign lesions or to characterize the tumor microenvironment in detail, and they ultimately require a tissue biopsy.

MRI, while offering superior soft tissue contrast and avoiding ionizing radiation, has traditionally faced challenges in lung imaging due to susceptibility effects from air-tissue interfaces, low proton density in the lungs, and respiratory and cardiac motion artifacts. These limitations have historically relegated lung MRI to a secondary role. However, recent advancements in MR hardware, pulse sequences, and motion correction techniques have begun to overcome some of these barriers, opening new avenues for the application of advanced MRI techniques such as CEST.

In this context, CEST MRI holds particular promise by offering molecular-level information about tumor physiology, such as extracellular pH, protein synthesis, and metabolic reprogramming - features that are not easily accessible through conventional imaging. This molecular insight could not only enhance early diagnosis and subtyping of lung tumors but also improve treatment monitoring by detecting subtle changes in tumor biochemistry in response to therapy.

This narrative review explores the principles, current applications, and future potential of CEST MRI in the context of lung malignancies, with a focus on clinical aspects and thoracic imaging. Emphasis is placed on its ability to probe tumor acidosis, protein concentration, and the broader tumor microenvironment, as well as on its emerging role as a functional biomarker for cancer diagnosis and management.

Background

The initial evaluation for suspected lung cancer typically involves a chest radiograph, which can reveal the presence of lung nodules or masses. However, due to its limited sensitivity and specificity, further imaging is usually required for definitive diagnosis and staging. Contrast-enhanced CT scan has become the mainstay for primary lung cancer diagnosis and staging, offering high spatial resolution and the ability to characterize lesions based on size, morphology, and contrast enhancement patterns. CT is particularly valuable for detecting small nodules, assessing local invasion, and evaluating lymph node involvement. Lung cancer is broadly classified into small-cell and non-small-cell varieties, as the specific diagnosis affects treatment modalities, with small-cell variants showing a better response to chemotherapy, while surgery remains a mainstay of treatment for non-small-cell lung cancer [7].

PET/CT plays a crucial role in the staging of lung cancer, particularly for evaluating distant metastasis and assessing the metabolic activity of primary tumors and lymph nodes [8]. The combination of functional (PET) and anatomical (CT) information provides valuable insights into the extent and aggressiveness of the disease, as the presence or absence of metastatic lesions influences treatment decisions.

In contrast to CT and PET/CT, conventional MRI has historically played a limited role in the primary diagnosis of lung malignancies. This is primarily due to the inherent technical challenges of lung MRI, most notably motion artifacts arising from respiration and cardiac activity, as well as susceptibility artifacts at the air-tissue interfaces, which can significantly degrade image quality and obscure subtle lung parenchyma details [4,5].

However, it's important to note that whole-body MRI has demonstrated superiority over PET/CT in the detection of specific distant metastases, particularly in the brain and liver, due to its higher soft tissue contrast and lack of ionizing radiation [8]. This highlights the potential of MRI in specific aspects of lung cancer management beyond primary diagnosis.

Despite the limitations of conventional MRI in primary lung imaging, CEST MRI is emerging as a promising technique that may offer unique advantages in this challenging area. By providing information about the tumor microenvironment and biochemical processes at a molecular level, CEST MRI has the potential to overcome some of the limitations of conventional anatomical imaging and provide valuable insights into lung cancer diagnosis, characterization, and treatment monitoring, as will be discussed in the subsequent sections.

## Review

Principles used in CEST MRI imaging for lung malignancies

Magnetization Transfer (MT) and Magnetization Transfer Ratio (MTR)

The basis of MRI relies upon the magnetic properties of protons (hydrogen nuclei) in nuclei and fat for conventional MRI imaging. Of note is that free protons in water tend to emit most of the magnetic signal used in MRI. Protons bound to large molecules, such as fats or proteins, which are common in the human body, are usually not detected via conventional MRI. Free protons and those bound to macromolecules do not exist in stasis, and instead are exchanged between these pools until equilibrium is achieved [9].

Coupling between the protons of these two pools - those free in water and those bound to large molecules - allows the protons bound to large molecules to influence the spin state of protons that are in water. The protons within the macromolecules can be preferentially saturated using an off-resonance RF pulse, not used with typical MRI imaging, as they are more sensitive to the off-resonant RF by a factor of as much as 10^6^ when compared to the free protons. The frequency needed for such is expressed in the form of parts per million (ppm), rather than the traditional unit for frequency, hertz, as the ppm shift is constant regardless of the strength of the magnetic field being used, which can vary across scanners.

ppm is an expression of the shift of the frequency compared to a baseline, where the frequency at which protons within the free water pool resonate is considered to be zero. The most commonly used off-resonant RF, 3.5 ppm, corresponds to the resonance of amide protons (-NH groups) found in most peptides and proteins.

This saturation is then transferred to the free protons within the water, and the rate of this saturation transfer is dependent upon the rate of exchange between protons in the two groups. The decrease in the signal from the bound protons can be measured over time. This MT can be read; however, due to practical concerns (mainly time and instrument limitations), the MTR is instead used.

MTR is calculated using two RF pulses - one that is off-resonant and one that is resonant (during which the free pool of protons is excited). The classical image taken without the off-resonant RF (expressed as M_0 _or S_0_) is then compared to the image taken with the off-resonant RF (expressed as M_S _or S_MT_) and calculated as:

$\mathrm{MTR} = \frac{M_0 - M_S}{M_0} \times 100$

In healthy tissue with a large number of macromolecules, after an off-resonant RF is applied, there is a strong MT, which disrupts the magnetization of the protons in the free pool. This results in a small M_S_ and subsequently a larger numerator (M_0 _- M_S_) while the inverse is true.

MTR showed potential when it came to characterizing lesions of multiple sclerosis (MS) as the loss of myelin because of MS and the presence of water from inflammation around the myelin sheath made it so that the disruption was less significant, M_S_ was larger, and hence a smaller ratio was seen in MS lesions [10]. Here, however, we will focus on the use of MTR as applied to the imaging of tumor cells.

Classical MTR measures overall saturation transfer. But it is not frequency specific and nor does it isolate chemical change effects.

While conventional MT and CEST MRI are mechanistically related, they represent distinct contrast mechanisms and should not be considered equivalent. Classical MT arises from saturation of a broad, semi-solid macromolecular proton pool (e.g., proteins and lipids) and is typically quantified using the MTR, which reflects non-specific interactions between macromolecules and bulk water. This is based on several factors, including chemical exchange from specific molecules (the aspect of interest), but also direct water saturation, macromolecular MT, and nuclear Overhauser effects (NOEs). NOEs are changes in the intensity of a nucleus’s resonance when a nearby nucleus is saturated with an RF field.

In contrast, CEST MRI relies on frequency-selective saturation of exchangeable solute protons (such as amide, amine, or hydroxyl protons) that resonate at specific offsets from water and undergo chemical exchange with bulk water. In order to isolate this aspect from confounding factors, advanced modeling methods are used to separate these overlapping contributions from one another.

The simplest model is the MTR asymmetry model, in which signal loss is compared at both a positive frequency and an equivalent negative frequency, with the underlying assumption that background effects are roughly symmetric around the water resonance, so subtracting cancels them out.

It is expressed as:

$\mathrm{MTR}_{\mathrm{asym}}(\Delta \omega) = \frac{S(-\Delta \omega) - S(+\Delta \omega)}{S_0}$

Here, S(-Δω) is the signal at a negative frequency offset, S(+Δω) is the signal at the positive frequency offset, and S_0 _is the control signal without saturation. This method is simple, fast, and widely accepted when it comes to clinical imaging. It has limitations, however, mainly that the underlying assumption that background effects are roughly symmetric around water resonance is oftentimes not true within certain tissues.

There are other advanced fitting models, such as Lorentzian line-shape fitting and Bloch-McConnell modeling, used to quantify CEST imaging, which are more complex and currently less practical for routine clinical use.

Conventional MRI uses gadolinium as a contrast material. While some CEST MRI techniques do not rely on exogenous contrast, others use contrast and its interaction with biological substances to enhance imaging. Because these contrast techniques are dependent upon certain metabolic processes - those which vary between cancer cells and normal cells - they can be used to differentiate malignant lesions from benign ones. Two major processes have been used as the basis for CEST MRI techniques: measurement of in vivo pH and protein synthesis.

pH-Sensitive Techniques

The normal pH of the human body typically remains between 7.35 and 7.45 [11]. Maintaining an optimal pH is essential for several physiological processes and biochemical reactions involved in homeostasis. The bicarbonate system is the most plentiful buffer within the human body to regulate pH [11-13]. The pH in tumor cells, however, tends to be more acidic, with studies showing ranges typically between 7.0 and 7.2 [13].

The pathology of tumor cells involves poor perfusion around the tumor area, as well as an acidic environment due to increased metabolism, primarily of glucose into lactate for the purposes of tumor proliferation. The physiological buffer within the body can deal with small changes in pH, and the vasculature can slowly wash away some of the metabolites, but this process is heightened in tumor cells to the point where the body's regulatory mechanisms cannot fully adapt, leading to an acidic microenvironment around the tumor cells.

Additionally, evidence suggests that not only does cancer metabolism lead to an acidic microenvironment, but the evolution of cells resistant to a more acidic environment is a crucial component of cancer pathogenesis. Cells that are able to adapt to this acidic microenvironment have a powerful growth advantage, which promotes the uncontrolled and unregulated growth typical of malignant cells. The optimal extracellular pH for cancer cell growth has been shown to be closer to 6.8, rather than the body's typical pH range. Because of this, in vivo pH measurement is a powerful diagnostic tool to identify malignancies. Additionally, as more aggressive tumor cells tend to tolerate a lower pH and are more resistant to therapy, this can also be used to evaluate patients and stratify them based on the potential efficacy of therapy. Targeting areas of low pH also has therapeutic applications, in addition to diagnostic ones, as certain pharmaceuticals are developed to target cells within this acidic microenvironment.

The most accurate measure of pH would be direct measurement of the tissue involved, but this is invasive and, therefore, impractical for potential clinical use in humans. Given this, the potential for in vivo measurement using various imaging modalities has been explored over the past years, with CEST MRI showing exceptional promise compared to alternative methods used previously, as described below.

PET scans using dimethadione, which distributes based on pH gradients across semipermeable membranes because of ion trapping, have been used since the 1970s in order to attempt to measure in vivo pH, but were inaccurate, as they depended upon the plasmalemmal pH gradient and the fractional volumes of intracellular and extracellular space, which cannot be easily calculated [14]. Because of this, the application of PET in measuring pH was limited.

Phosphorus nuclear magnetic resonance spectroscopy was used to estimate intracerebral pH in rabbits and rats as early as 1985 [15-17]. While this could be used to obtain estimations of pH, it was limited in attempting to visualize tumors, due to the fact that detailed structures and their relationships to each other could not be visualized using spectroscopy alone, limiting its application for locating tumors for later biopsy if needed.

It is here that CEST MRI has shown promise. Part of the usefulness of CEST MRI in lung malignancies lies in its sensitivity to changes in pH within the tumor microenvironment. The chemical exchange rate of labile protons, which forms the basis of CEST contrast, is intrinsically linked to pH. Specifically, the exchange rate of many relevant functional groups (like amide and amine protons) increases under alkaline conditions and decreases in acidic environments, due to the base-catalyzed nature of the exchange process [18,19]. Because of this altered exchange rate, more acidic environments appear different on MRI imaging and are more easily visualized in terms of their spatial relation with other structures, facilitating later tissue biopsy if needed. This pH sensitivity allows CEST MRI techniques to indirectly or directly probe the acidic environment often associated with tumor metabolism. Different CEST MRI techniques have been developed based on the principle of in vivo pH measurement, and are described below.

APT MRI for pH estimation: APT imaging, a widely used CEST technique, leverages the exchange of amide protons (-NH) from endogenous mobile proteins and peptides with bulk water to estimate pH. Amide protons resonate at a signal intensity of 3.5 ppm. While APT signal intensity is primarily influenced by the concentration and mobility of these proteins, the rate of proton transfer is also significantly dependent on pH within the physiological range. In an acidic environment, the exchange rate slows down, potentially leading to lower APT signal intensity, although the relationship is complex and influenced by other factors, such as, but not limited to, protein concentrations, temperature, and the presence of other macromolecules [18-20]. From this, MTR values can be calculated for differing tissues. One major benefit of APT is that it is contrast-free, with the amide proteins themselves acting as “contrast” to generate differing images.

The observation that tumor cells often exhibit increased glycolytic metabolism (the Warburg effect), leading to the production of lactic acid and a more acidic extracellular pH, makes APT MRI a potentially valuable tool in oncology [21]. By detecting these subtle pH variations non-invasively, APT MRI could aid in various aspects of lung malignancy diagnosis and treatment, as outlined below.

Studies like the one by Zhou et al. demonstrated the ability of APT imaging to detect pH changes in acute stroke due to lactic acidosis [22]. Following a stroke, blood supply to the area is compromised, leading to an acidic environment and a change in pH. This study highlighted APT MRI's sensitivity to pH shifts in vivo.

Many of the studies regarding the viability of APT MRI to detect pH differences have been done on brain malignancies, owing to the easier technical aspects of brain imaging via MRI. MRI has been used for the diagnosis of gliomas, and APT signal intensity has been associated with survival and progression in high-grade glioma patients [23-26]. Additionally, associations have been found between APT signal intensity and glioma expression of the isocitrate dehydrogenase genes (IDH1 and IDH2), which are involved in cellular metabolism [27]. IDH1 and IDH2 expression is important to identify, as prognosis differs, as well as treatment regimens, and expression of these genes is a target for newer drugs such as vorasidenib.

Applying this to lung tumors, Ohno et al. showed significantly higher MTR_asym​ values in malignant lung lesions compared to benign ones in human patients, suggesting that APT MRI might provide a contrast mechanism related to the altered pH or protein content within tumors, which have a lower pH than ordinary cells and also a higher rate of proliferation [6]. Furthermore, they observed differences in MTR values between adenocarcinoma and squamous cell carcinoma subtypes, hinting at the potential for histological characterization without the need for invasive biopsy.

In patients with brain metastasis from lung cancer, CEST MRI can be used without the typical drawback of imaging difficulties seen in obtaining thoracic MRI images, in order to characterize lung malignancies, as demonstrated by Xiang et al. [26]. They were able to predict, based on imaging, whether the primary lung cancer was squamous cell carcinoma or adenocarcinoma.

Changes in tumor pH in response to therapy could potentially be tracked using APT MRI. For example, treatments that aim to normalize tumor metabolism or improve vascularization might lead to changes in the APT signal, as has been demonstrated in studies on brain tumors [28,29].

The major drawback of APT MRI for pH estimation is the presence of confounding factors, the most significant of which is the presence of mobile peptides. These peptides can increase the APT signal, as they are highly water-accessible. The acidic pH, on the other hand, decreases APT signals, as described above. Most tumor cells have increased mobile peptides as part of tumor metabolism and pathogenesis, so, in certain cases, these two factors can confound measurement, and certain tumors, such as gliosarcomas in rats, have shown no difference in APT signal. While APT MRI does have the benefit of not requiring any kind of external contrast, there are limitations to its usage.

AcidoCEST MRI for direct pH measurement: To overcome the indirect pH sensitivity of APT, acidoCEST MRI has emerged as an alternative. AcidoCEST MRI utilizes exogenous contrast agents, such as iopamidol, whose amide proton exchange rates with water are more directly and predictably dependent on pH, and less affected by confounding factors [30,31]. By selectively saturating the protons of the injected contrast agent and measuring the resulting signal change in water, acidoCEST can provide higher specificity for measuring pH, though it is still dependent on agent concentration and perfusion clearance kinetics.

A study comparing the measurement of pH via acidoCEST MRI to values obtained via a pH microsensor in mouse models showed the accuracy of acidoCEST MRI in pH measurement.

As highlighted by Lindeman et al. [32], acidoCEST MRI showed a lack of strong correlation with APT-measured pH, suggesting that it provides a different, and potentially more direct, measure of the extracellular environment. Lindeman et al.'s [33] work in mice demonstrated the potential of acidoCEST MRI to differentiate between different types of lung lesions based on their pH profiles, which could be crucial in distinguishing malignancy from inflammatory or infectious conditions, which also have more acidic pH environments.

While the usage of contrast enhances the accuracy of measurement, iopamidol contrast is not without its own risks. Common side effects noted after administration of iopamidol include mild allergic reactions, such as hives and nausea, but can also include more serious adverse events, such as anaphylaxis, contrast-induced nephropathy, seizures, and arrhythmias, though these are extremely rare [34]. In patients who have had prior adverse reactions, pretreatment with antihistamines and steroids is often considered, but such patients may still have serious adverse events [35].

In summary, both APT MRI and acidoCEST MRI offer pH-based approaches for investigating lung malignancies. APT MRI, relying on endogenous proteins, is label-free but provides an indirect measure of pH, influenced by protein concentration. AcidoCEST MRI, using exogenous agents, offers a more direct assessment of extracellular pH, but faces challenges related to contrast agent administration and safety. Future research needs to further explore and validate both techniques in human lung cancer patients to determine their clinical utility in diagnosis, characterization, and monitoring of the disease.

Protein-Based CEST Imaging

Beyond pH-based measurements, CEST MRI can also offer tools for imaging based on proteins. Tumor cells, for their unregulated proliferation and growth, require increased synthesis of peptides. This technique capitalizes on the fact that cancer cells often exhibit altered metabolism and increased proliferation rates, leading to an upregulation in the synthesis of various proteins and peptides involved in cell growth, survival, and invasion [36,37]. The increased concentration of these endogenous, mobile proteins and peptides provides an inherent contrast mechanism detectable by APT MRI.

It is here that APT MRI imaging finds use beyond the measurement of pH. A study of brain metastasis showed that 66% of the observed APT signal was due to protein concentration alterations, and 34% of it was due to differences in pH, though this percentage is dependent on the context of the method used [38].

Use of APT methods with regard to lung imaging is less well documented in the literature; however, Yang et al. [39] used this method to differentiate between gliomas and metastatic brain lesions from lung malignancies in human patients and those without disease. APT imaging was used for this approach and found significant imaging differences between gliomas and metastatic brain lesions from lung malignancies.

As an alternative to APT, synthetic peptides have been developed in order to facilitate CEST MRI. Compounds such as salicylic acid possess CEST signals far from ordinary tissue background, and release a strong signal that can be detected via CEST MRI. Synthetic peptides, which are then cleaved or metabolized into compounds that release a strong signal under CEST MRI, have been used for drug release monitoring and targeting in inflammatory bowel disease [40].

Synthetic peptides can be used for tumor imaging either through peptides that tag specific substrates, genetically encoded proteins that are substrate-independent, or enzymes that manipulate CEST contrast in order to generate substrates that can be detected via CEST MRI [41,42].

Peptides can be synthetically generated, organic, and biodegradable. Hydroxyl, guanidyl, amine, and amide groups can be incorporated, which can be excited at specific RFs. These peptides can be used either directly or indirectly by monitoring their changes under specific cellular environments.

Genetically encoded reporters work by manipulating genetic expression within cells. One of the first attempts involved encoding a synthetic gene that expressed a lysine-rich peptide. These genes can be inserted via viruses into cells, and their expression monitored via CEST MRI.

Potential clinical applications​​​​​​​

Non-invasive Differentiation of Benign and Malignant Lesions and Histopathological Classification

CEST MRI, particularly through pH-sensitive (APT, acidoCEST) and protein-sensitive (APT) contrasts, has the potential to improve the accuracy of distinguishing malignant lung nodules from benign conditions. Additionally, it can be used to differentiate between histopathological subsets of tumors. This could aid in personalized treatment planning. Further research correlating CEST parameters with histopathological findings is needed. CEST MRI identifies more aggressive tumors, as well as gene expression within tumor cells, which can also be useful in prognosis and treatment planning.

This could reduce the need for invasive biopsies in a subset of patients with indeterminate lesions detected on conventional imaging, leading to earlier diagnosis and reduced patient anxiety. Future studies with larger cohorts and direct comparisons to standard diagnostic pathways are crucial to validate this potential.​​​​​​​

Guiding Therapeutic Efforts

CEST MRI can potentially be used to grade more aggressive tumors by detecting the microenvironment around the tumor cells, thereby guiding prognosis and optimizing treatment regimens. CEST MRI can also be used to detect genomic expression in certain cancer cells, as demonstrated with IDH1 and IDH2 expression, as mentioned above.

Several chemotherapeutic agents are affected by pH environments. Adriamycin, a widely used chemotherapeutic agent, shows decreased efficacy in acidic environments [43]. Adriamycin itself is acidic and, therefore, shows decreased permeability across cell membranes in acidic environments; evaluation of such pH environments of tumor cells before the commencement of therapy can help better tailor treatment regimens. 

Early Biomarker of Therapy Efficacy

CEST MRI's sensitivity to metabolic changes and cellular proliferation could provide an early indication of treatment response to various therapies, including chemotherapy, radiotherapy, targeted therapy, and immunotherapy. Detecting changes in CEST parameters (e.g., APT signal and pH maps) earlier than changes in tumor size on anatomical imaging could allow for timely adjustments in treatment strategies. A study comparing CEST MRI and FDG-PET/CT for predicting the therapeutic effect of chemoradiotherapy (CRT) on stage III non-small cell lung cancer (NSCLC) patients showed that CEST MRI has potential for predicting the effect of CRT [44]. Longitudinal studies evaluating CEST MRI during and after different therapies are essential to further explore this area.

Differentiation of True Response From Pseudo-Progression

In the context of immunotherapy, CEST MRI might help differentiate true tumor progression from pseudo-progression (an initial increase in tumor size due to immune cell infiltration). Changes in the tumor microenvironment or cellularity, as assessed by CEST, could provide additional information beyond size criteria.

Assessment of Treatment-Induced Changes in Tumor Microenvironment

CEST MRI could be used to monitor how therapies alter the tumor microenvironment, such as changes in pH or metabolic activity, which might provide insights into the mechanisms of resistance or sensitivity.

Early Detection of Recurrence

CEST MRI could potentially play a role in the early detection of local recurrence or distant metastasis after primary treatment. Changes in pH or protein content in suspicious areas, identified on surveillance imaging, could prompt further investigation before they would be detectable on conventional imaging.

Limitations of CEST MRI in lung imaging​​​​​​​

Reliability of APT-Based pH Measurement

As highlighted by some studies, the APT signal is influenced by both protein concentration and pH, making direct and reliable pH quantification challenging. Advanced quantification techniques and careful validation are necessary to overcome this limitation. The development and validation of more specific pH-sensitive CEST agents (acidoCEST or endogenous alternatives) are crucial; however, these contrast agents come with their own drawbacks.

Safety and Practicality of Exogenous Contrast Agents (acidoCEST)

The use of intravenous contrast agents, like iopamidol, in acidoCEST MRI raises concerns regarding potential nephrotoxicity, allergic reactions, and the logistical challenges of contrast administration. Further research into safer and more easily cleared exogenous CEST agents, or the development of robust endogenous pH-sensitive CEST methods, is needed.​​​​​​​

Respiratory and Cardiac Motion Artifacts

Motion remains a significant impediment to high-quality CEST MRI of the lungs. While techniques like respiratory gating, triggering, and advanced post-processing methods (e.g., 4D reconstruction, as mentioned by Eiben et al. [45]) are being explored, robust and clinically feasible solutions that consistently provide high-resolution, motion-free CEST images are still needed [45,46]. This is particularly important for quantitative analysis of CEST parameters. One partial solution noted above is in patients with malignant spread of primary lung malignancies to areas that are easier to image, such as the brain, for differentiating cancer subgroups. 

Tumor Inflow Bronchi

Virtual bronchoscopy, using CT-guided reconstruction, is widely used for the detection of bronchial branches of vessels that provide blood flow to tumors, in order to guide planning for tissue biopsy [47]. Virtual bronchoscopic navigation greatly facilitates not only diagnosis, but also potentially therapeutic resection of lung lesions [48-50]. Currently, there have been no studies on the use of CEST-MRI techniques as a comparison or adjunct to CT-guided reconstruction, which is a potential area for future exploration.​​​​​​​

Limited Studies in Human Patients

A significant portion of the current research has been conducted in preclinical animal models. While these studies provide valuable insights into the biological basis and technical feasibility of CEST MRI, translation to human studies is essential to validate its clinical utility in lung cancer diagnosis and management. Larger, well-designed clinical trials are required.​​​​​​​

Standardization and Reproducibility

The lack of standardized acquisition protocols and post-processing methods across different research centers hinders the direct comparison of results and the implementation of CEST MRI in multi-center clinical trials. Efforts toward standardization are crucial for ensuring the reproducibility and generalizability of CEST MRI findings.​​​​​​​

Long Acquisition Times

CEST MRI sequences can be relatively long, which can be problematic for patients, especially those with respiratory compromise. Developing faster acquisition techniques, without compromising image quality and CEST contrast, is an ongoing challenge.

The various techniques used in CEST MRI imaging - their applications, advantages, and limitations - are summarized in Table 1.

**Table 1:** Summarizing Various Techniques Used in CEST MRI CEST MRI, Chemical Exchange Saturation Transfer Magnetic Resonance Imaging

CEST MRI Technique	Overall Advantages	Key Limitations
Amide Proton Transfer (APT) MRI	1. Uses endogenous mobile proteins and peptides as intrinsic contrast, eliminating the need for exogenous contrast agents.	1. The indirect measurement of pH is confounded by variations in protein and peptide concentration, temperature, and tissue composition.
	2. Sensitive to tumor-related changes in protein concentration and extracellular pH, reflecting increased cellular proliferation and altered metabolism.	2. Increased mobile peptides in tumors can mask pH-related signal changes, further reducing the reliability of quantitative pH estimation.
	3. Has demonstrated potential for differentiating malignant from benign lung lesions and for histopathological subtype characterization.	3. Susceptible to motion artifacts and technical challenges in lung imaging, and clinical validation in large human cohorts remains limited.
	4. Enables early assessment of treatment response by detecting biochemical changes prior to anatomical alterations.	-
AcidoCEST MRI	1. Allows more direct and predictable measurement of extracellular tumor pH using exogenous contrast agents with well-characterized pH-dependent exchange properties.	1. Requires intravenous administration of contrast agents such as iopamidol, which introduces risks including allergic reactions, nephrotoxicity, and logistical constraints.
	2. Reduces confounding effects seen in endogenous techniques and improves the accuracy of pH mapping.	2. Still subject to motion artifacts in thoracic imaging and limited by the availability of standardized protocols and large-scale human studies.
	3. Shows promise for distinguishing malignant lesions from inflammatory or infectious processes and for characterizing tumor aggressiveness.	-
Protein-Based CEST Imaging (Endogenous)	1. Exploits increased protein and peptide synthesis associated with tumor proliferation to generate intrinsic contrast.	1. Protein-based contrast cannot be fully separated from pH effects in endogenous techniques, complicating interpretation.
	2. Provides insight into tumor cellularity and metabolic activity.	2. Limited clinical data exist for lung malignancies, and signal specificity may vary across tumor types and microenvironments.
	3. Can complement pH-based imaging to improve lesion characterization and assessment of aggressiveness.	-
Synthetic Peptide and Targeted CEST Agents	1. Enables highly specific molecular targeting, including enzyme activity, drug delivery, and gene expression.	1. Requires exogenous agents or genetic manipulation, raising safety, regulatory, and translational challenges.
	2. Produces strong CEST signals at frequencies distinct from background tissue, improving detectability.	2. Primarily investigated in preclinical models. Clinical feasibility and safety in lung cancer patients remain unproven.
	3. Offers potential for combined therapeutic and diagnostic (sometimes called “theranostic”) applications and treatment monitoring.	-
Genetically Encoded CEST Reporters	1. Allows non-invasive monitoring of gene expression and cellular behavior using MRI.	1. Currently limited to experimental and animal studies.
	2. Provides highly specific molecular information independent of endogenous tissue variability.	2. Requires genetic modification of target cells, making clinical application impractical at present.

## Conclusions

CEST MRI represents a transformative approach to lung cancer imaging, offering a non-invasive window into the biochemical complexities of the tumor microenvironment. By leveraging pH- and protein-sensitive mechanisms, such as APT and acidoCEST, this technique provides unique metabolic insights that complement traditional anatomical modalities.

However, widespread clinical translation requires addressing significant technical hurdles, including the mitigation of respiratory motion artifacts and the standardization of acquisition protocols. Future efforts must prioritize large-scale clinical validation and protocol harmonization to fully integrate CEST MRI as a diagnostic tool. 
